# Constraints on GPCR Heterodimerization Revealed by the Type-4 Induced-Association BRET Assay

**DOI:** 10.1016/j.bpj.2018.09.034

**Published:** 2018-11-22

**Authors:** James H. Felce, Alasdair MacRae, Simon J. Davis

**Affiliations:** 1 MRC Human Immunology Unit, MRC Weatherall Institute of Molecular Medicine, University of Oxford, Oxford, OX3 9DS, United Kingdom; 2 Radcliffe Department of Medicine, University of Oxford, Oxford, OX3 9DU, United Kingdom

## Abstract

G-protein-coupled receptors (GPCRs) comprise the largest and most pharmacologically important family of cell-surface receptors encoded by the human genome. In many instances, the distinct signaling behavior of certain GPCRs has been explained in terms of the formation of heteromers with, for example, distinct signaling properties and allosteric cross-regulation. Confirmation of this has, however, been limited by the paucity of reliable methods for probing heteromeric GPCR interactions in situ. The most widely used assays for GPCR stoichiometry, based on resonance energy transfer, are unsuited to reporting heteromeric interactions. Here, we describe a targeted bioluminescence resonance energy transfer (BRET) assay, called type-4 BRET, which detects both homo- and heteromeric interactions using induced multimerization of protomers within such complexes, at constant expression. Using type-4 BRET assays, we investigate heterodimerization among known GPCR homodimers: the CXC chemokine receptor 4 and sphingosine-1-phosphate receptors. We observe that CXC chemokine receptor 4 and sphingosine-1-phosphate receptors can form heterodimers with GPCRs from their immediate subfamilies but not with more distantly related receptors. We also show that heterodimerization appears to disrupt homodimeric interactions, suggesting the sharing of interfaces. Broadly, these observations indicate that heterodimerization results from the divergence of homodimeric receptors and will therefore likely be restricted to closely related homodimeric GPCRs.

## Introduction

Comprising >700 proteins, G-protein-coupled receptors (GPCRs) are the largest family of cell-surface receptors encoded by the human genome and are of great pharmacological importance. The essential features of GPCR signaling have been well characterized over the last >40 years; however a number of key questions remain unanswered. The issue of GPCR stoichiometry, particularly for the largest GPCR subfamily—the rhodopsin receptors—is still a matter of some debate, with arguments both for and against the formation of receptor homo-oligomers. Of particular pharmacological interest is the extent to which GPCRs also heterodimerize, as different combinations of paired receptors could generate druggable complexes with unique signaling properties (1). Whether such complexes exist is also contentious, however (2, 3, 4), despite the large number of studies claiming receptor heteromerization for a variety of receptors (5).

Leaving aside the problem that apparent cooperative behavior between GPCRs could be due to receptor cross talk rather than heteromerization (6), the key technical issue is that the great majority of the studies proposing heterodimerization were performed using coimmunoprecipitation (co-IP), single-point resonance energy transfer (RET), or bioluminescence RET (BRET) saturation assays, the interpretation of which can be problematic. Briefly, co-IP is complicated because it is inherently biased toward the detection of oligomers because monomers cannot be detected and also because the extent of receptor association that might be induced during solubilization per se is an unknown. RET assays, which have the advantage of reporting stoichiometry in situ, exploit the nonradiative transfer of energy from a donor molecule to an acceptor fluorophore, which typically only occurs if the two molecules are within ∼10 nm of one another. However, single-point RET assays do not usually account for nonspecific RET between membrane proteins, (i.e., that occurring between noninteracting proteins). The difficulty of discriminating between nonspecific and specific RET has been a considerable challenge, and several approaches have been proposed to overcome it. The widely used BRET saturation assay involves the measurement of BRET efficiency (BRET_*eff*_) at a range of acceptor concentrations and constant donor density, but the interpretation of this assay is complicated by the varying levels of nonspecific RET resulting from changes in acceptor density alone (7). This is particularly problematic in the case of heterodimerization because differences in expression rates or subcellular distribution of receptor subunits increases the likelihood of data deviating from pseudolinearity and therefore the risk of reporting false dimers. Arguably, the most powerful tool for examining receptor stoichiometry, that of single-molecule imaging, has yet to be used to demonstrate GPCR heterodimerization, even though it is largely responsible for the present consensus that homodimerization is generally transient and the dominant GPCR stoichiometry is that of monomers (8). Single-molecule spectroscopic techniques, such as fluorescence cross-correlation spectroscopy, have reported heterodimerization in a number of cases (9), but these approaches have yet to become routine.

GPCR heteromer-identification technology (HIT) is a BRET-based approach designed to identify GPCR heterodimers (10). In GPCR-HIT, receptor A is fused to the donor luciferase (Rluc), whereas receptor B is untagged. Acceptor fluorophores are expressed as fusions with appropriate signaling proteins, typically arrestins. The acceptor is recruited to receptor B upon agonist binding, giving increased BRET_*eff*_ if the receptors are interacting, as reported for several GPCR pairs (11, 12, 13). No increase is expected for noninteracting proteins, but it has been argued elsewhere (7) that the recruitment of cytosolic arrestin to the plasma membrane will increase nonspecific BRET through an increase in effective acceptor concentration. Moreover, some GPCRs and their signaling partners do not necessarily experience free diffusion (14, 15) or uniform membrane distribution (16, 17, 18, 19); this further complicates interpretation because the recruitment of acceptor to a genuine donor-containing heteromer versus a region of the cell surface simply enriched for donors cannot be distinguished.

Three previously proposed BRET assays (types 1–3), which reliably report receptor homodimerization, are not well suited to the investigation of heterodimerization. Types -1 and -2 BRET assays (20) rely on equivalent rates of protein expression from acceptor- and donor-tagged constructs for a range of acceptor/donor ratios or total densities, which cannot be guaranteed when expressing heterodimers. The type-3 competition assay (21) could be used to identify heterodimers but only with caveats. First, this assay would only identify heterodimers formed by proteins that also homodimerize; otherwise, competitors have no effect on BRET_*eff*_. Second, heterodimers that do not disrupt homodimerization (e.g., heterodimers of homodimers) would also be undetectable because homodimeric complexes, and hence BRET_*eff*_, would be unaffected by the presence of a competitor. Finally, changes in BRET_*eff*_ would also be attributable to factors independent of heterodimerization, such as signaling cross talk or depletion of a common interaction partner. Presently, there is no BRET-based approach that unambiguously identifies GPCR heteromers.

Here, we describe a BRET-based assay for membrane protein heterodimerization based on the induced multimerization of one of the interaction partners at constant expression. We benchmark the assay using the known GPCR homodimers, CXC chemokine receptor 4 (CXCR4) and the sphingosine-1-phosphate (S1P) receptors and observe that heterodimerization occurs in a manner that disrupts homodimer formation.

## Materials and Methods

### Cloning of constructs for expression of transfected proteins

Details of the strategy used to generate green fluorescent protein (GFP)-tagged proteins have been published previously (22). The vector used for this, pGFP^2^, was used as the template for the generation of all expression vectors used in this study to ensure equivalent expression across constructs. A vector containing FK506-binding protein (*FKBP*) in place of *GFP*^*2*^ was generated from the pGFP^2^ vector by PCR amplification of *FKBP* from the pC_4_-F_V_1E vector (Ariad, Cambridge, MA) using oligonucleotide primers 1 and 6 (Data S1). This produced an *FKBP* fragment with 5′ *BamHI* and 3′ *NotI* restriction sites as well as a TAG STOP codon after the final codon of *FKBP* and a Gly-Ser-Gly-Ser-Gly-encoding linker between the *BamHI* site and the start of *FKBP*. This fragment was used to replace *GFP*^*2*^ in pGFP^2^ by ligation after digestion with *BamHI* and *NotI* to remove *GFP*^*2*^.

To generate constructs tagged with three FKBP proteins (separated by flexible linkers of Gly-Ser-Gly-Ser-Gly), *FKBP*_*1*_ was removed from the above vector and replaced by *FKBP*_*3*_. The *FKBP*_*3*_ fragment was generated by PCR amplification of *FKBP* from the pC_4_-F_V_1E vector (Ariad) using oligonucleotide primer pairs 1 + 2, 3 + 4, and 5 + 6 (Data S1). This yielded three copies of *FKBP* with the following 5′ and 3′ restriction sites: *BamHI* + *SacI*, *SacI* + *XhoI*, and *XhoI* + *NotI*. All copies were preceded by the sequence GGTTCCGGATCGGGG, encoding the flexible linker, and the final copy was followed by a TAG STOP codon. These three copies were sequentially ligated together after digestion with *SacI* and *XhoI* to generate a complete *FKBP*_*3*_ fragment with *BamHI* and *NotI* 5′ and 3′ restriction sites. This was then used to replace *FKBP*_*1*_ in the aforementioned vector by ligation after *BamHI* and *NotI* digestion.

Luciferase 8 (Rluc8) was used in place of conventional Rluc because of its greatly increased brightness and stability. *Rluc8* was generated by site-directed mutagenesis of *Rluc* amplified from the pRluc vector using PCR. This was performed in a series of chimeric reactions to generate the following mutations: A55T, C124A, S130A, K136R, A143M, M185V, M253L, and S287L, using oligonucleotide primers listed in Data S1. This was then used to replace *GFP*^*2*^ in pGFP^2^ by ligation after digestion with restriction endonucleases *BamHI* + *EcoRI*. The integrity of all vectors was confirmed by reversible terminator base sequencing. All necessary genes were then subcloned into each vector using appropriate restriction sites within the multiple-cloning site of the pGFP^2^ vector.

To generate a positive control suitable for use in type-4 BRET, a soluble GFP^2^-Rluc8 construct was produced by PCR amplification of *GFP*^*2*^ from pGFP^2^ using oligonucleotide primers 20 and 21 (Data S1). This generated a *GFP*^*2*^ gene with a 5′ *MluI* restriction site and Kozak sequence and a 3′ *BamHI* site. This was then ligated into the pRluc8 vector after digestion with *MluI* and *BamHI* and confirmed for integrity using reversible terminator base sequencing. The resulting vector encoded a soluble GFP^2^ protein fused to Rluc8 via its C-terminus.

### Type-4 BRET assay

**Figure 1:**
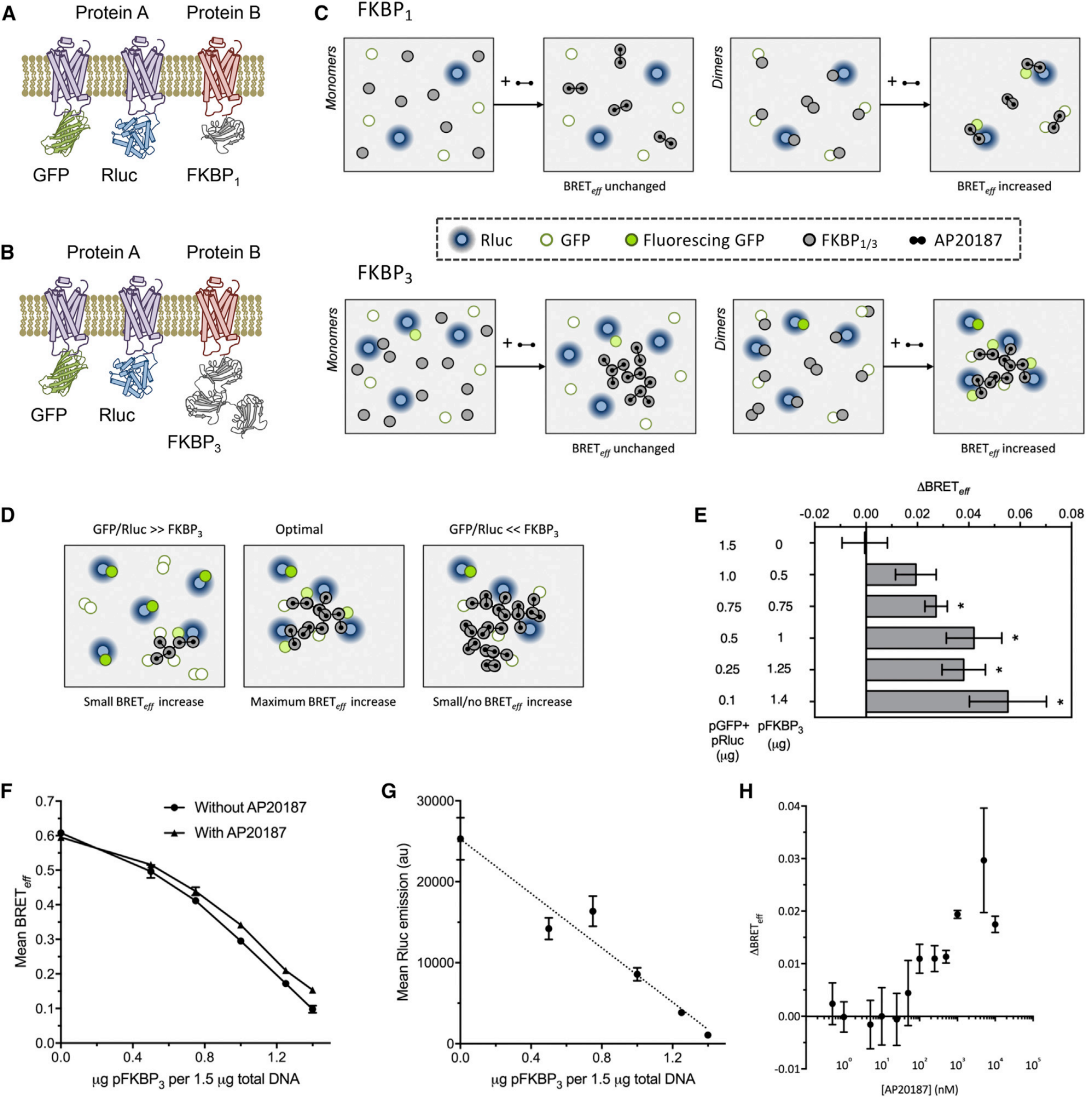
Principle and optimization of the type-4 BRET assay. (*A*) The expression strategy for type-4 BRET; protein A is expressed as a typical GFP-/Rluc-tagged BRET pair, whereas protein B is tagged with FKBP to allow ligand-induced dimerization. (*B*) Protein B can also be tagged with three sequential copies of FKBP to allow ligand-induced oligomerization. (*C*) Addition of the bivalent ligand AP20187 induces dimerization of FKBP_1_ (*top*) and oligomerization of FKBP_3_ (*bottom*). If proteins A and B are interacting, this will lead to corecruitment of GFP and Rluc molecules and hence increase BRET_*eff*_; however, the distribution of monomeric GFP-/Rluc-tagged proteins will be unaffected. (*D*) The extent of BRET_*eff*_ increase on AP2017 ligation is sensitive to the ratio of GFP-/Rluc- versus FKBP-tagged proteins. A large excess of GFP/Rluc over FKBP (*left*) will allow only minimal recruitment of GFP/Rluc on induced association. Conversely, a large excess of FKBP (*right*) will result in recruited GFP/Rluc remaining widely separated in the induced multimer. (*E*) Measured *Δ*BRET_*eff*_ values for CD28 using various ratios of the pFKBP_3_ and pGFP/pRluc vectors; ^∗^*p* < 0.05. (*F*) Absolute mean BRET_*eff*_ values for CD28 at different ratios pFKBP_3_ and pGFP/pRluc with and without AP20187. (*G*) The mean Rluc emission for CD28-Rluc at different ratios pFKBP_3_ and pGFP/pRluc; the dotted line shows a linear regression of points. (*H*) Measured *Δ*BRET_*eff*_ for CD28 as a function of AP20187 concentration used to induce association, using a constant 1 *μ*g pFKBP_3_ and 0.5 *μ*g pGFP/pRluc,. For all panels, bars represent mean ± SD. All data are the result of *n* = 3 independent experiments.

Transfection for the type-4 assay was performed in six-well plates containing ∼6 × 10^5^ human embryonic kidney (HEK) 293T cells per well. Cells were transfected with 1.5 *μ*g total DNA per well using the GeneJuice (Novagen, Madison, WI) transfection reagent per the manufacturer’s protocol. Unless otherwise stated, this DNA was a mix of pFKBP_1/3_, pGFP^2^, and pRluc8 in a ratio of 26:12:1. The ratio of pFKBP_1/3_:pGFP^2^/pRluc8 of 2:1 was optimized experimentally (Fig. 1 *E*). The 12:1 ratio of pGFP^2^/pRluc8 was used to ensure an excess of GFP^2^ over Rluc8 because this provides maximal BRET_*eff*_ and also minimizes variation in BRET_*eff*_ caused by slight differences in absolute GFP^2^/Rluc8 between replicate experiments. 24 h post transfection, cells were removed from six-well plates and suspended in 200 *μ*L phosphate-buffered saline (PBS). This was separated into 2 × 100 *μ*L in two wells of a 96-well OptiPlate (PerkinElmer, Waltham, MA). To one well, 10 *μ*L AP20187 was added to 5 *μ*M (unless alternative concentration is specified), whereas 10 *μ*L PBS was added to the other. Cells were then incubated for 45 min at room temperature before data collection. BRET_*eff*_ was measured as described previously (21). Briefly, emission from transfected cells incubated with the Rluc8 substrate coelenterazine 400A was measured at two wavelengths corresponding to direct Rluc8 emission (410 ± 401 nm) and RET-dependent GFP^2^ emission (515 ± 15 nm) and normalized to the BRET_*eff*_ measured for GFP^2^-Rluc8 positive control. All data shown are the result of at least three independent experiments.

Data obtained by type-4 analysis were analyzed using the Prism5 (GraphPad, La Jolla, CA) software. Data were treated as paired sets corresponding to the measurements taken in the presence and absence of AP29187 for a single well of transfected cells, thereby generating a *Δ*BRET_*eff*_ value for each transfection. These were compared to *Δ*BRET_*eff*_ values obtained for cells expressing only GFP/Rluc8-tagged proteins to control for nonspecific effects of AP20187. “Normalized” *Δ*BRET_*eff*_ values were hence calculated as actual *Δ*BRET_*eff*_ minus the *Δ*BRET_*eff*_ in the absence of FKBP (Eq. 1). Significance was determined using a two-tailed *t*-test to evaluate whether the mean *Δ*BRET_*eff*_ between the FKBP+ and FKBP− measurements were significantly different.

$$\text{Normalized}\;\Delta {\text{BRET}}_{eff}\;=\Delta {\text{BRET}}_{eff}^{with\;FKBP}\;-\;\Delta {\text{BRET}}_{eff}^{without\;FKBP}\;\;\;$$

and

(1) $$\Delta {\text{BRET}}_{eff}\;={\text{BRET}}_{eff}^{with\;AP20187}\;-\;{\text{BRET}}_{eff}^{without\;AP20187}\text{.}$$

### Types-1, -2, and -3 BRET assays

The types-1, -2, and -3 BRET assays were performed using previously published protocols (20, 21).

### Confocal microscopy

Surface expression of CD28 in the presence and absence of AP20187 was assessed via confocal microscopy. HEK-293T cells were transfected in an identical manner to that used for type-4 analysis. Cells were incubated with 5 *μ*M AP20187 or PBS for 45 min at room temperature before fixation using PBS + 4% paraformaldehyde for 10 min. Imaging was performed using a Zeiss LSM-510 inverted confocal microscope (Carl Zeiss AG, Oberkochen, Germany) under an oil-immersed 60× objective lens with excitation laser light at 488 and a 515 ± 15 nm emission filter. Images were collected at the midpoint of the cell and minimally manipulated to improve the signal-to-noise ratio.

### Flow cytometry

Flow cytometry used to determine relative construct expression was performed on CD2-GFP, CD2-FKBP_1_, CD2-FKBP_3_, and CD2-Rluc. HEK-293T cells were transfected with 1 *μ*g of the relevant vector per 6 × 10^5^ cells using GeneJuice (Novagen) per the manufacturer’s protocol. 24 h post transfection, cells were labeled for flow cytometry analysis using 0.05 *μ*g/*μ*L of either mouse anti-human CD2 antibody (12-0029-42; eBioscience, San Diego, CA) or mouse IgG isotype control (12-4714-12; eBioscience). Cells were then assessed using a CyAn ADP (Beckman Coulter, Brea, CA) flow cytometer for fluorescence in the phycoerythrin channel.

## Results

### The type-4 BRET assay

In the type-4 BRET assay, putative interaction partners (proteins A and B) are coexpressed in the same cells: protein A in the form of a Rluc- and GFP-tagged BRET pair and protein B fused to FKBP (Fig. 1 *A*). FKBP is induced to homodimerize using the bivalent ligand AP20187. BRET_*eff*_ is measured in both the presence and absence of AP20187; if there is no interaction between A and B, induced dimerization of B has no effect on BRET_*eff*_ because the distribution of A is unaffected (Fig. 1 *C*). Conversely, if the two proteins interact, dimerization of B increases the effective concentration of acceptor around each donor by indirectly recruiting A. Thus, BRET_*eff*_ increases for heterodimers. Higher-order hetero-oligomerization should also be detectable using type-4 BRET because induced protein-B dimerization should increase acceptor abundance around each donor. The type-4 BRET principle can be used in two ways: by inducing dimerization of protein B via a single FKBP tag (FKBP_1_; Fig. 1 *A*) or inducing oligomerization using, for example, three sequential FKBP tags (FKBP_3_; Fig. 1 *B*), which would increase the signal/noise ratio. Moreover, the GFP/Rluc:FKBP ratio can be varied. Maximal *Δ*BRET_*eff*_ (i.e., the difference between BRET_*eff*_ in the presence and absence of AP20187) is obtained when FKBP-tagged protein is in excess to maximize interactions with GFP-/Rluc-tagged proteins, but expression is not so high as to preclude BRET-productive oligomer formation upon addition of AP20187 (Fig. 1 *D*).

To optimize the new assay, type-4 BRET was performed in HEK-293T cells transfected with the well-characterized covalent dimer, the type-1 transmembrane protein CD28, at varying ratios of pFKBP_3_ and pGFP/pRluc DNA, using a total of 1.5 *μ*g DNA per 6 × 10^5^ cells. *Δ*BRET_*eff*_ varied with the ratio used (Fig. 1 *E*). No increase in BRET_*eff*_ was observed when only pGFP/pRluc was used, indicating that AP20187 does not have FKBP-independent effects on BRET_*eff*_. Positive *Δ*BRET_*eff*_ values were always obtained when pFKBP_3_ was included, although this was only statistically significant above a value of 0.75 *μ*g pFKBP_3_ per transfection. *Δ*BRET_*eff*_ did not alter significantly above a level of 1 *μ*g pFKBP_3_ per transfection despite a reduction in absolute mean BRET_*eff*_ in both the presence and absence of AP20187 at higher pFKBP_3_ levels (Fig. 1 *F*) because of competition by untagged pFKBP_3_ within homodimers as well as at the level of translation as in the case of Rluc (Fig. 1 *G*). At 1.4 *μ*g pFKPB_3_, total Rluc emission in a typical measurement is ∼1000 AU. At ∼500 AU, accurate BRET_*eff*_ determination becomes difficult as the level of “bleed-through” from the donor to acceptor channels (∼2% total Rluc emission) is comparable to the background signal of the assay (∼10 AU). In light of this, and the above considerations regarding *Δ*BRET_*eff*_, we selected, as an optimal transfection strategy, 1 *μ*g pFKBP_3_ and 0.5 *μ*g pGFP/pRlu8. We also established the concentration of AP20187 that maximized *Δ*BRET_*eff*_. Too little AP20187 will leave some FKBP molecules unligated, whereas too much might saturate the FKBP proteins, preventing oligomerization. Titration of AP20187 revealed that 5 *μ*M AP20187 yielded maximal *Δ*BRET_*eff*_ for CD28 (Fig. 1 *H*).

### Proof of concept that type-4 BRET reports protein homo- and heterodimerization

**Figure 2:**
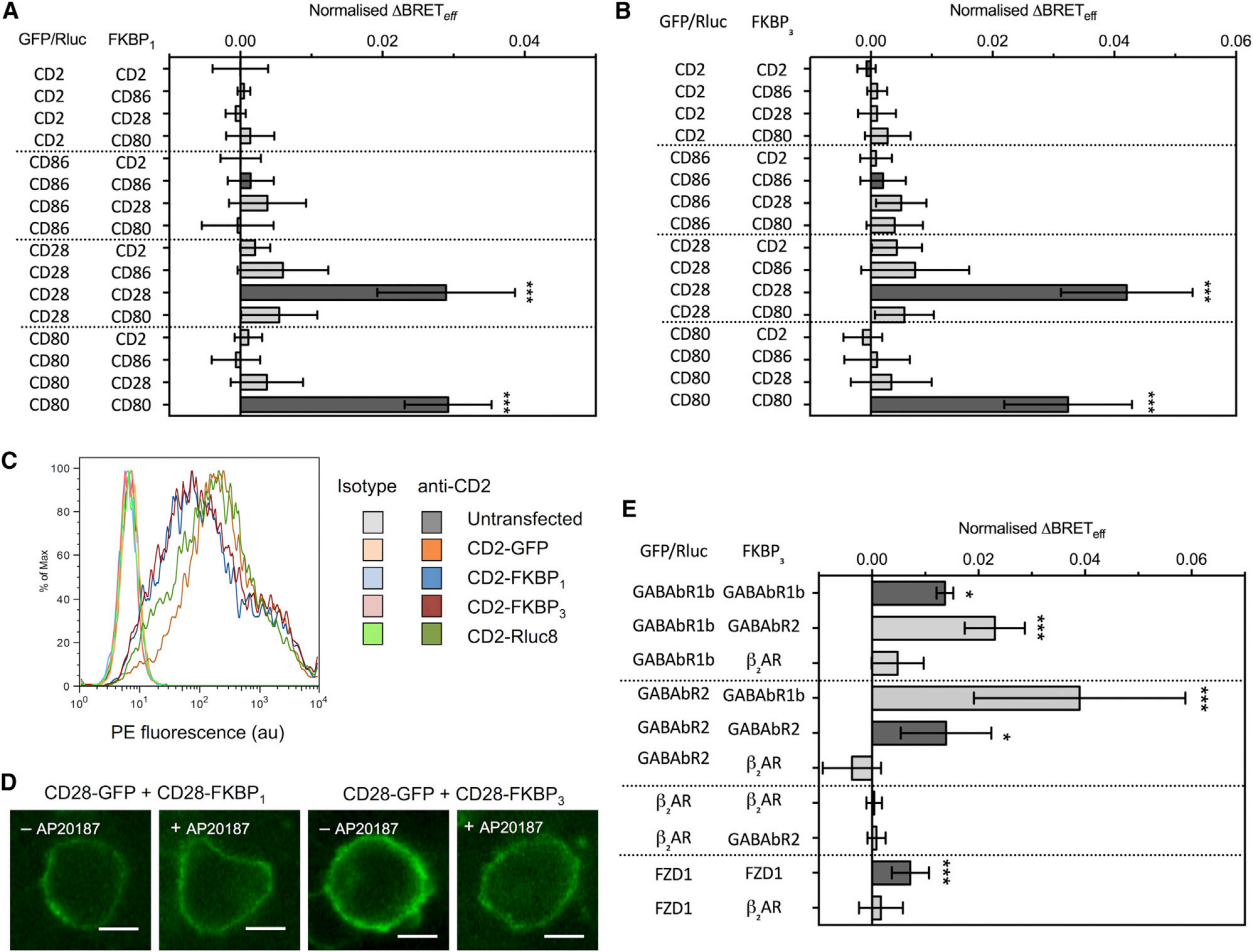
Type-4 BRET assay applied to known controls. (*A*) The mean *Δ*BRET_*eff*_ of type-1 transmembrane protein controls using the FKBP_1_ type-4 BRET assay. Only the known dimers CD28 and CD80 exhibited significantly nonzero *Δ*BRET_*eff*_ when expressed as homomeric pairs. Dark gray bars indicate like-like pairs. (*B*) The mean *Δ*BRET_*eff*_ of type-1 transmembrane protein controls using the FKBP_3_ type-4 BRET assay. *Δ*BRET_*eff*_ is larger for CD28 and CD80 than with the FKBP_1_ approach. (*C*) A histogram of CD2 expression in HEK-293T cells transfected with 1 *μ*g of differently tagged forms of CD2, as measured by flow cytometry. (*D*) Confocal microscopy of CD28-GFP coexpressed with CD28-FKBP_1/3_ in the presence and absence of AP20187. No clear GFP internalization was evident. (*E*) The mean *Δ*BRET_*eff*_ of GPCRs of known stoichiometry using the FKBP_3_ type-4 BRET assay. For all panels, bars indicate mean ± SD. Probability is indicated for difference from *Δ*BRET_*eff*_ = 0; ^∗^*p* < 0.05, ^∗∗∗^*p* < 0.005. All data are the result of *n* ≥ 3 independent experiments.

We next sought to show that the type-4 method identifies dimers and to determine which strategy, utilizing FKBP_1_ or FKBP_3_, yielded the greatest sensitivity. We did this by analyzing type-1 transmembrane proteins of known stoichiometry. Cells were transfected with a 2:1 ratio of pFKBP_1/3_:pGFP/pRluc and a 12:1 ratio of pGFP/pRluc—i.e*.*, 26:12:1 pFKBP_1/3_:pGFP:pRluc. Each culture was split into two separate wells of a 96-well plate for BRET_*eff*_ measurement, to which either PBS or 5 *μ*M AP20187 (final concentration) was added. Measurement of BRET_*eff*_ for these two wells after 45 min of incubation at room temperature generated paired data sets in which cells were equivalent except in the presence or absence of AP20187. All data were normalized to *Δ*BRET_*eff*_ values collected in cells expressing only the GFP- and Rluc-tagged proteins. For the known monomers CD2 and CD86, no significant *Δ*BRET_*eff*_ was observed regardless of which protein comprised the FKBP_1_- or FKBP_3_-tagged partner (Fig. 2, *A* and *B*). Conversely, the known dimers CD28 and CD80 both exhibited significant *Δ*BRET_*eff*_ but only when the homodimeric GFP/Rluc-FKBP pair was used, confirming the specificity of the assay. Whereas CD28 is a covalent homodimer (23), CD80 dimerizes in a transient and noncovalent manner (24); thus, the type-4 BRET method also identifies partial dimers. The mean normalized *Δ*BRET_*eff*_ observed for CD28 using FKBP_3_ (0.042) was substantially larger than that observed using FKBP_1_ (0.029), and CD80 also exhibited a modest improvement from 0.029 to 0.033. The apparent noise in the measurements for the two approaches was similar (mean coefficient of variation for CD28 and CD80 of 27.2% for FKBP_1_ and 29.1% for FKBP_3_), meaning that the increased mean normalized *Δ*BRET_*eff*_ was also associated with increased lower 95% confidence limits in the FKBP_3_ system (e.g., 0.018 for CD28-FKBP_1_ and 0.031 for CD28-FKBP_3_). The differences in FKBP_1_ and FKBP_3_ reporting were not due to differences in expression efficiency because this was comparable (Fig. 2 *C*). It could not be attributed to AP20187-induced protein internalization because no such effect was evident according to confocal microscopy of HEK-293T cells expressing CD28-GFP and either CD28-FKBP_1_ or CD28-FKBP_3_ (Fig. 2 *D*).

The utility of the type-4 method was also validated using a known GPCR heterodimer. The *γ*-amino butyric acid receptors GABAbR1b and GABAbR2 form homo- and heterodimers (25) and produced significantly increased BRET_*eff*_ in the assay using FKBP_3_-tagged versions of either receptor but not of the unrelated *β*_2_ adrenergic receptor (*β*_2_AR; Fig. 2 *E*). Interestingly, both GABAb receptors yielded greater *Δ*BRET_*eff*_ values when expressed as the heteromeric pair than as homodimers. *β*_2_AR did not give significant increases in BRET_*eff*_ with either itself or GABAbR2 as the FKBP_3_-tagged partner, consistent with our previous observations of monomeric behavior (20, 21, 26), whereas Frizzled-1, confirmed previously to be dimeric (22), yielded a significant increase (Fig. 2 *E*), further confirming the ability of the type-4 assay to identify GPCR homo- and heterodimers.

### The chemokine receptors CCR5 and CXCR4 demonstrate contrasting stoichiometries yet form heterodimers

**Figure 3:**
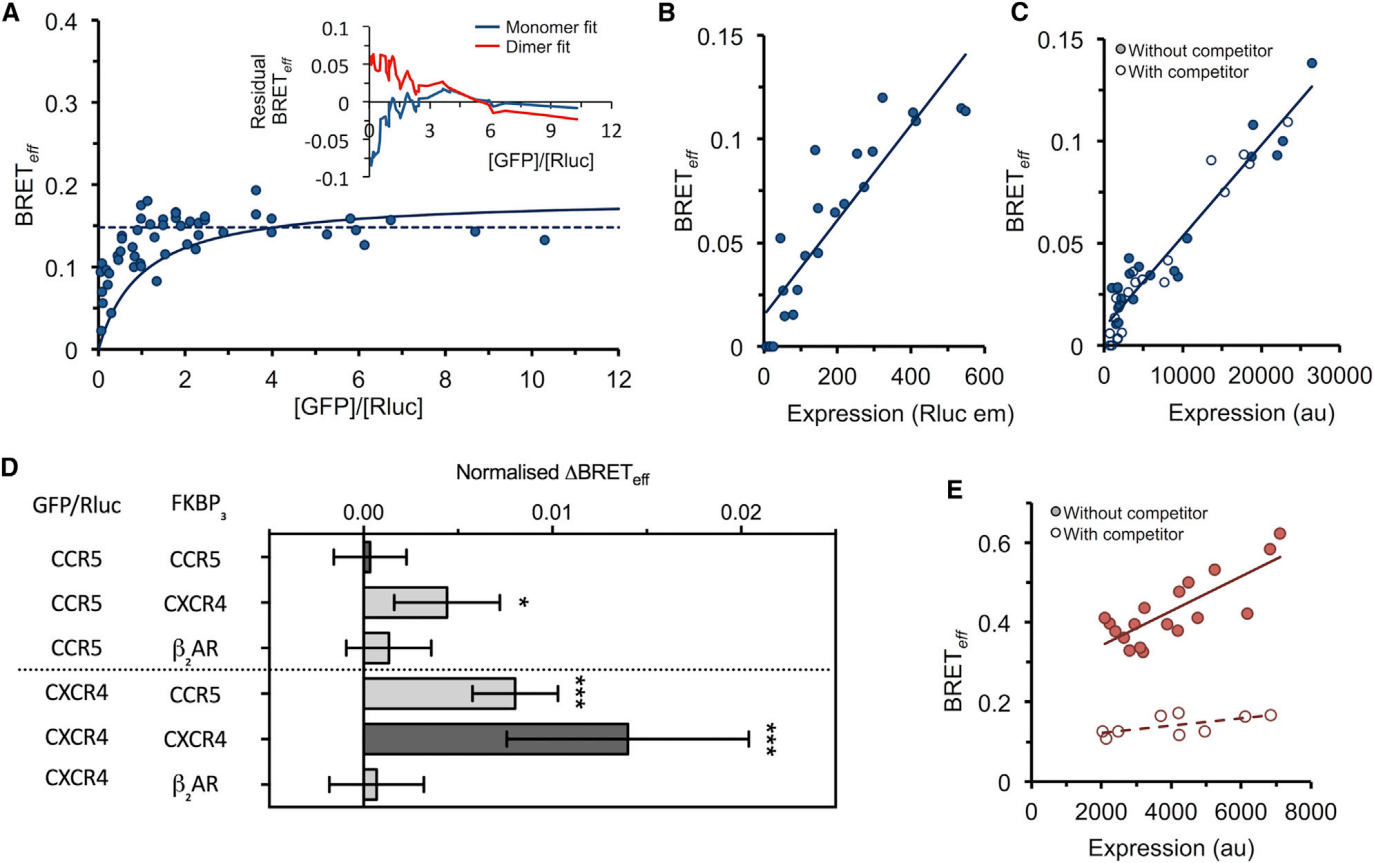
The chemokine receptors CCR5 and CXCR4 form heterodimers, but CCR5 does not homodimerize. (*A*) Type-1 BRET of CCR5 suggests monomeric behavior. BRET_*eff*_ is independent of [GFP]/[Rluc] above a value of ∼2, consistent with the relationship exhibited by monomers. Fits of the data to models of monomeric (*broken line*) and dimeric (*solid line*) behavior are shown, and the residual values (*inset*) are shown as a moving average (*n* = 4). Only residual values above [GFP]/[Rluc] = 2 are considered when determining stoichiometry. (*B*) Type-2 BRET analysis of CCR5 also indicates monomericity as the *y*-intercept of linear regression (*solid line*) of BRET_*eff*_ versus expression level is not significantly nonzero. (*C*) Type-3 BRET of CCR5 confirms monomeric behavior. The BRET_*eff*_-expression relationship is equivalent in the presence and absence of untagged competitors. Linear regression of both data sets combined is shown as a solid line. (*D*) Type-4 BRET analysis of CCR5 and CXCR4 paired with themselves, each other, and *β*_2_AR. Both CCR5 and CXCR4 exhibited significant *Δ*BRET_*eff*_ when paired with each other, but only CXCR4 yielded significant *Δ*BRET_*eff*_ when paired with itself. Bars represent mean ± SD; ^∗^*p* < 0.05, ^∗∗∗^*p* < 0.005. (*E*) Type-3 BRET of CXCR4 in the presence and absence of untagged CCR5 competitors reveals disruption of CXCR4 homodimers by CCR5. All data are the result of *n* = 3 independent experiments.

The CXC chemokine receptor 4 (CXCR4) is within the subset of rhodopsin-family GPCRs comprising substantive homodimers (22). We sought to test the suggestion (27) that CXCR4 can heterodimerize with the closely related CC-motif chemokine receptor 5 (CCR5). We first characterized the capacity of CCR5 to homodimerize using type-1,-2, and -3 BRET assays (20, 21). In the type-1 assay, BRET_*eff*_ for dimers exhibits a hyperbolic dependence on acceptor/donor ratio, whereas for monomers, it is independent of acceptor/donor ratio above a value of ∼2. CCR5 exhibited monomeric behavior in this assay (Fig. 3 *A*), in contrast to CXCR4 (22). The type-2, and -3 assays also reported monomeric behavior for CCR5 (Fig. 3, *B* and *C*). Briefly, in the type-2 assay, acceptor/donor ratio is kept constant while total density is varied, resulting in BRET_*eff*_ for monomers tending to zero at low densities, as observed for CCR5 (Fig. 3 *B*). In the type-3 assay, the BRET_*eff*_-density relationship is measured in the presence and absence of untagged “competitor” proteins, which reduce BRET_*eff*_ at a given concentration for dimers and oligomers but not for monomers, as observed for CCR5 (Fig. 3 *C*). The low *Δ*BRET_*eff*_ obtained for CCR5 in the type-4 assay (Fig. 3 *D*) also suggested monomeric behavior. When paired with CXCR4-FKBP_3_, CCR5 exhibited a small but significant positive *Δ*BRET_*eff*_, suggesting the existence of CCR5-CXCR4 heterodimers. Similarly, the CXCR4-GFP/Rluc:CCR5-FKBP_3_ pair also exhibited enhanced *Δ*BRET_*eff*_. The mean *Δ*BRET_*eff*_ for this pair was smaller than that for CXCR4-GFP/Rluc:CXCR4-FKBP_3_, which may indicate that CXCR4 homodimers are more stable than CXCR4-CCR5 complexes, although the difference is not statistically significant and might have arisen by chance only.

To test whether CCR5 influences CXCR4 homodimerization, we performed a type-3 BRET assay (21) of CXCR4 heterodimerization in the presence and absence of untagged CCR5 competitors. This reported a significant decrease in BRET_*eff*_ in the presence of CCR5, suggesting that CCR5 disrupts CXCR4 homodimerization. The simplest explanation for this is that the CCR5-CXCR4 interaction precludes simultaneous CXCR4 homodimerization at a shared or partially shared interaction interface (suggested for CXCR4-CXCR4 to be formed by transmembrane helices 3, 4, and 5 (28)), but other explanations are possible (e.g., the interaction with CCR5 alters the conformation of CXCR4, preventing homodimerization).

### The S1P receptors exhibit varying strengths of heterodimerization

**Figure 4:**
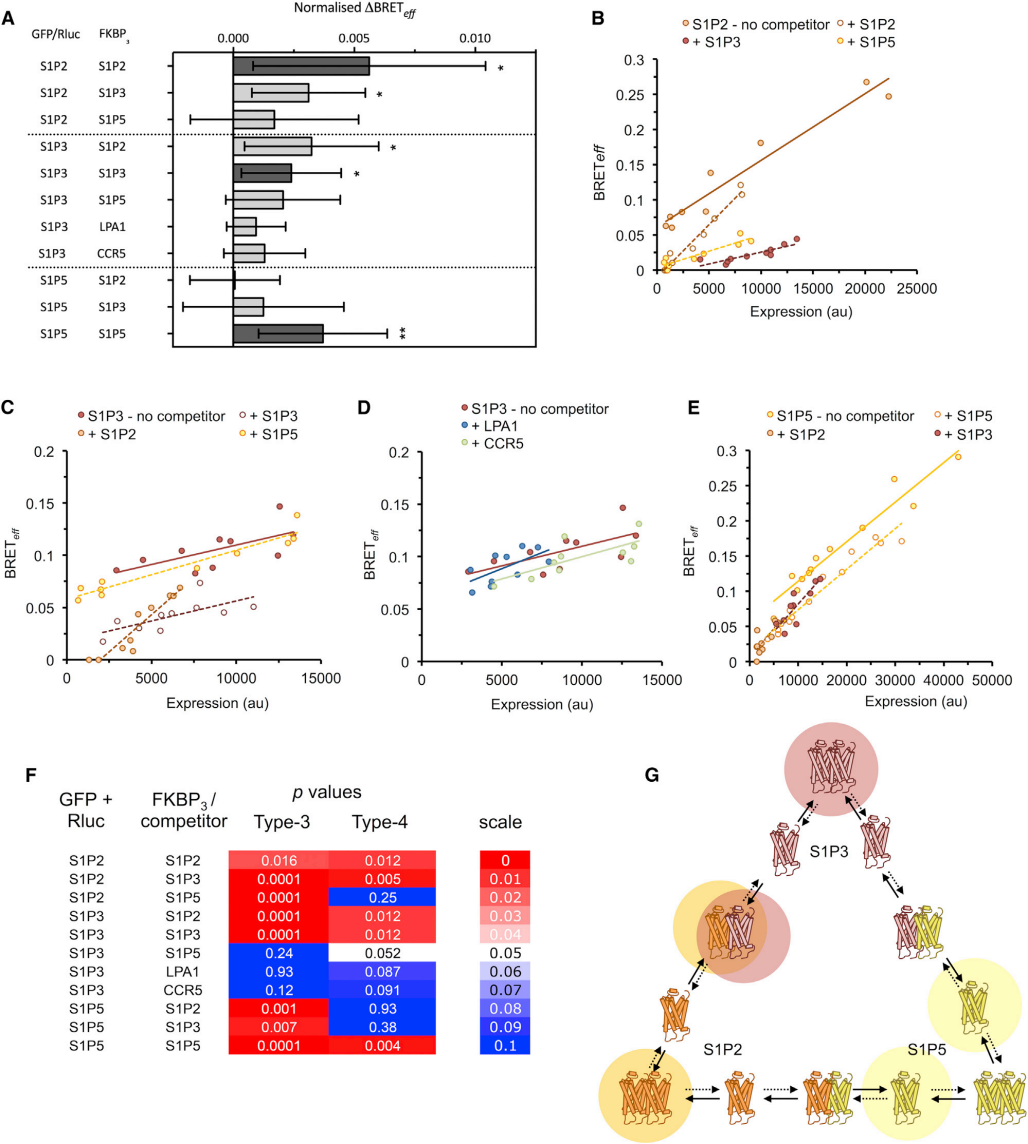
The S1P receptors heterodimerize with different strengths of association. (*A*) Type-4 BRET analysis of S1P receptors in various combinations and of S1P3 with LPA1 and CCR5. All three S1P receptors yielded significant *Δ*BRET_*eff*_ as homomeric pairs, as did S1P2 and S1P3 when paired together. Bars represent mean ± SD; ^∗^*p* < 0.05, ^∗∗^*p* < 0.01. (*B*) Type-3 BRET data of S1P2 with either S1P2, 3, or 5 as untagged competitors. Significant reduction in BRET_*eff*_ versus expression was observed with all three competitors, indicating heteromerization. Linear regression of data in the presence and absence of competitors is shown as broken and solid lines, respectively. Type-3 analysis of S1P3 revealed significant reductions in BRET_*eff*_ in the presence of S1P2 but not S1P5 (*C*) or LPA1 or CCR5 (*D*). (*E*) S1P5 exhibited reduced BRET_*eff*_ with all three S1P receptors in the type-3 assay. (*F*) Summarized statistics for the S1P receptors under the types-3 and -4 BRET assays. Values are color coded according to the scale, in which red denotes *p* < 0.05 and blue denotes *p* > 0.05. (*G*) A possible interaction scheme of S1P receptors. S1P2 is represented in orange, S1P3 in red, and S1P5 in yellow. Broken arrows indicate a slow transition; solid arrows represent a fast transition. The predominant species of each receptor are highlighted in the relevant color of that receptor. In this model, the S1P2 and S1P3 receptors are largely homodimers or in heterodimeric complexes with one another, whereas the S1P5 is more frequently monomeric, and dimerization is restricted chiefly to homodimerization. All data are the result of *n* ≥ 3 independent experiments.

Other candidates for heterodimerization are the S1P receptors (22), which bind the lysophospholipid S1P (29) and are involved in neuronal and vascular development (30) and leukocyte migration (31). Humans possess five S1P receptors, three of which (S1P2, 3, and 5) behave as homodimers in types-1 and -3 assays (22). Interestingly, the closely related lysophosphatidic acid (LPA) receptors, which are often grouped with the S1P receptors as the “endothelial differentiation” GPCR subfamily, behave exclusively as monomers in these assays (22), indicating a high degree of specificity in the interaction mediating S1P dimerization. S1P2, S1P3, and S1P5 exhibited significant increases in BRET_*eff*_ in the type-4 BRET assay when tested as homodimeric GFP/Rluc-FKBP_3_ pairs, consistent with the previous findings (Fig. 4 *A*; (22)). Importantly, increased *Δ*BRET_*eff*_ was also observed for S1P2 paired with S1P3 in both combinations, whereas neither S1P2 nor S1P3 exhibited increased BRET_*eff*_ in either combination with S1P5 (Fig. 4 *A*). S1P3 also failed to exhibit significantly increased *Δ*BRET_*eff*_ when paired with FKBP_3_-tagged CCR5 or LPA1.

Complementing these observations, we also performed type-3 BRET on all three S1P receptors using either the same or different receptors as competitors. S1P2 exhibited reduced BRET_*eff*_ with S1P2 and S1P3 competitors but also with S1P5 (Fig. 4, *B* and *F*). S1P3 only showed a significant reduction in BRET_*eff*_ in the presence of S1P2 or S1P3 competitors (Fig. 4, *C*, *D*, and *F*). S1P5 also exhibited a reduction in BRET_*eff*_ with competitors of all three S1P receptors, although most significantly with itself (Fig. 4, *E* and *F*). The discrepancies between the types-3 and -4 assays can be explained in two ways. First, S1P5 may only interact very weakly with S1P2 and S1P3 because the type-3 BRET assay is more sensitive than the type-4. Alternatively, there may be no genuine interaction between S1P5 and the other receptors, and instead, the presence of S1P5 alters the behavior of S1P2/3, reducing BRET_*eff*_ in the type-3 assay. We are presently unable to distinguish between these possibilities. The observation that, of the three S1P receptors investigated, S1P5 has the smallest decrease in BRET_*eff*_ upon addition of competitor in the homo-type-3 analysis (Fig. 4 *E*) nevertheless supports the notion that S1P5 has the lowest dimerization potential. These considerations suggest a tentative S1P interaction scheme (Fig. 4 *G*). S1P2 and S1P3 are proposed to be predominantly homo- or heterodimeric alone or with each other. S1P5, on the other hand, has a larger monomeric fraction but can homodimerize and interact with S1P2 and S1P3 at low levels. The dominant dimeric S1P5 state is that of homodimer.

## Discussion

GPCR heterodimerization, if it occurs widely, would have important implications for the functions of these receptors and for medicine. Many receptors have been proposed to form a broad array of different heteromeric complexes generating cooperative signaling effects (e.g., (32, 33)). In principle, however, in many cases, technical caveats and alternative explanations for the observed effects obviate the need for direct heteromeric interactions (6). Accordingly, whereas heterodimerization is wholly established for some glutamate family receptors (34, 35), beyond these examples, the case for GPCR heterodimerization is much more tentative. Our previous finding that homodimeric rhodopsin-family GPCRs, when they are observed, often involve closely related receptors (22) suggests that heterodimerization might be possible, mediated by very similar, shared interfaces. Here, we present a BRET assay designed explicitly to identify membrane protein heterodimers and use it to demonstrate heteromeric interactions among closely related rhodopsin-family GPCRs. Although the type-4 BRET assay reports heterodimerization, it also robustly identifies homodimers, complementing analyses using types-1, -2, and -3 BRET assays.

The key difference between type-4 BRET and other BRET approaches relying on induced association, such as GPCR-HIT (10) or third-party BRET (36), is that the acceptor- and donor-tagged proteins are not directly affected by the change imposed upon the system (i.e., addition of AP20187). In both GPCR-HIT and third-party BRET, the acceptor is inherently affected by the imposed change (i.e., arrestin recruitment for GPCR-HIT), complicating interpretation. Although increases in BRET_*eff*_ in the presence of AP20187 in type-4 experiments are readily explained by direct interaction between the GFP-/Rluc- and FKBP-tagged proteins, alternative explanations need also to be considered. First, the formation of large cross-linked oligomers might trap GFP- and Rluc-tagged proteins within corralled areas of the membrane, increasing BRET_*eff*_. However, this seems unlikely because corralling would be expected to affect the diffusion rather than the effective concentration of the trapped proteins. Evidence for this is that the observed behavior of monomers is equivalent in type-4 experiments with FKBP_3_ and FKBP_1_, and FKBP_1_ is much less prone to inducing cluster formation. A second possibility is that induced association of FKBP-tagged proteins causes their internalization, which might also nonspecifically increase BRET_*eff*_. Although this does not appear to apply to CD28 according to our confocal microscopy analysis, the effect could be more or less significant for other proteins depending on their mode of internalization. A third limitation of the type-4 BRET assay is that only diffusing heterodimers can be detected. If the GFP-/Rluc- or FKBP-tagged proteins are immobile, then BRET-productive induced dimers are unlikely to form.

A number of additional caveats need to be considered when interpreting the results of the type-4 assay. Firstly, the relative expression levels of the two proteins will have a profound impact on the outcome of the type 4 assay. If one receptor is expressed at significantly higher levels than the other, then the optimal ratio of GFP/Rluc:FKBP will not be achieved. The expression levels of the proteins investigated here have previously been quantified (22), and suitably matched GFP-/Rluc- and FKBP-tagged proteins were used. Secondly, although GPCRs with small C-terminal fusion tags such as GFP, Rluc, and FKBP are widely studied and considered to behave normally, the formal possibility exists that signaling may be affected in rare cases. Similarly, although unlikely, inducing oligomerization in the FKBP_3_-tagged receptors could change their capacity to heterodimerize with the GFP-/Rluc-tagged partners. The flexible linker between the C-terminus of the tagged protein and the FKBP tag should generally allow interaction interfaces to remain accessible to heteromeric partners. It is also possible that induced recruitment stabilizes homodimers because of the increased local concentration of similar receptors. In cases in which heterodimerization is competitive with homodimerization (as we observed for several receptor pairs), this would reduce the potential for heterodimerization. In such cases, BRET_*eff*_ would likely still increase on oligomerization of the FKBP-tagged protein because GFP- and Rluc-tagged proteins would be released from heterodimeric complexes and freed to form additional BRET-productive homodimers. The type-4 assay cannot distinguish between this effect and increased BRET_*eff*_ caused by the local concentration of GFP-/Rluc-tagged receptors upon induced oligomerization, but both would be a consequence of heterodimerization.

The type-4 BRET data obtained for CCR5 and CXCR4 are consistent with other results, suggesting such chemokine receptor heteromerization (27). T cells express CXCR4 constitutively but only express substantial amounts of CCR5 after activation by dendritic cells. The disruption of CXCR4 homodimerization by CCR5 may allow T cells to make migratory responses appropriate to their activation status. The observation that homodimerization is not required for heterodimerization in the case of CCR5 is intriguing because it suggests that the small fraction of homodimeric rhodopsin-family GPCRs (22) may be influenced by the more-numerous monomers. However, CXCR4 did not interact with other receptors generally, indicating that heterodimers may form mostly or entirely among closely related receptors at similar shared interfaces. Whether other monomers disrupt GPCR dimers requires more investigation.

The existence of S1P receptor heterodimers was suggested previously on the basis of *β*-galactosidase complementation (37) and co-IP (38) experiments, but the same investigations reached contradictory conclusions with regard to their heterodimerization with LPA receptors: S1P1-LPA1 interactions were reported using *β*-galactosidase complementation but not by co-IP. We have previously shown that S1P2, S1P3, and S1P5 behave as homodimers, whereas the closely related LPA1, LPA2, and LPA3 receptors do not (22). Heterodimerization within the S1P subfamily but not LPA receptors reflects a gain of dimerization after the divergence of the S1P and LPA subfamilies. This contrasts with the chemokine receptors, in which CCR5 blocked homodimerization of CXCR4. The S1P3 homodimer is in part stabilized by interactions involving transmembrane helix 4 (22), and it seems likely that S1P heterodimers use the same interface. S1P5 shares less transmembrane helix 4 sequence identity with S1P2 and S1P3 than they do with one another (S1P2-S1P5 = 48% identity, S1P3-S1P5 = 35% identity, and S1P2-S1P3 = 65% identity), perhaps explaining why S1P5 appears to form the weakest homo- and heterodimeric interactions.

But how widespread is heterodimerization among GPCRs? Our results suggest that, for the most part, heterodimerization may rely on an existing capacity for homodimerization*,* that is, that homodimers emerged first during receptor evolution and subsequently continued to interact after gene duplication and divergence. This is supported by the finding that we only detected heterodimers between closely related receptors and not between subfamilies. This suggests that GPCR heterodimerization, when it occurs, may be restricted to close relatives. This conflicts with the many reports of rhodopsin-family GPCR heterodimerization, often involving distantly related partners (e.g., (39, 40, 41)). Moreover, if homodimerization is generally a requirement for heterodimerization, then it seems likely that most rhodoposin-family GPCRs will not heterodimerize given the dominance of monomeric receptors within the family (22). Nonetheless, the example of CCR5 suggests that some capacity to heterodimerize might exist independently of homodimerization ability.

## Author Contributions

J.H.F. and S.J.D. conceived the problem and wrote the manuscript. J.H.F. designed the experimental strategy, performed the experiments, and analyzed the data. A.M. assisted with cloning, data collection, and optimization for the FKBP_1_ system.

## Supporting Material

Data S1. Oligonucleotide Primers Used in this Study
